# Effects of single- and mixed-bacterial inoculation on the colonization and assembly of endophytic communities in plant roots

**DOI:** 10.3389/fpls.2022.928367

**Published:** 2022-08-29

**Authors:** Ting Zhang, Juan Xiong, Rongchuan Tian, Ye Li, Qinyi Zhang, Ke Li, Xiaohong Xu, Lianming Liang, Yi Zheng, Baoyu Tian

**Affiliations:** ^1^The Provincial University Key Laboratory of Cellular Stress Response and Metabolic Regulation, College of Life Sciences, Fujian Normal University, Fuzhou, China; ^2^College of Biological Science and Engineering, Fuzhou University, Fuzhou, China; ^3^Library, Fujian Normal University, Fuzhou, China; ^4^Key Laboratory for Conservation and Utilization of Bio-resources, Yunnan University, Kunming, China

**Keywords:** plant endophytic microbiome, probiotics, bacterial inoculation, bacterial colonization, lignocellulase

## Abstract

The introduction and inoculation of beneficial bacteria in plants have consistently been considered as one of the most important ways to improve plant health and production. However, the effects of bacterial inoculation on the community assembly and composition of the root endophytic microbiome remain largely unknown. In this study, 55 strains were randomly isolated from tomato roots and then inoculated into wheat seeds singly or in combination. Most of the isolated bacterial strains showed an ability to produce lignocellulose-decomposing enzymes and promote plant growth. The results demonstrated that bacterial inoculation had a significant effect on the wheat root endophytic microbiome. The wheat root samples inoculated with single-bacterial species were significantly separated into two groups (A and B) that had different community structures and compositions. Among these, root endophytic communities for most wheat samples inoculated with a single-bacterial strain (Group A) were predominated by one or several bacterial species, mainly belonging to *Enterobacterales*. In contrast, only a few of the root samples inoculated with a single-bacterial strain (Group B) harbored a rich bacterial flora with relatively high bacterial diversity. However, wheat roots inoculated with a mixed bacterial complex were colonized by a more diverse and abundant bacterial flora, which was mainly composed of *Enterobacterales*, *Actinomycetales, Bacillales, Pseudomonadales*, and *Rhizobiales*. The results demonstrated that inoculation with bacterial complexes could help plants establish more balanced and beneficial endophytic communities. In most cases, bacterial inoculation does not result in successful colonization by the target bacterium in wheat roots. However, bacterial inoculation consistently had a significant effect on the root microbiome in plants. CAP analysis demonstrated that the variation in wheat root endophytic communities was significantly related to the taxonomic status and lignocellulose decomposition ability of the inoculated bacterial strain (*p* < 0.05). To reveal the role of lignocellulose degradation in shaping the root endophytic microbiome in wheat, four bacterial strains with different colonization abilities were selected for further transcriptome sequencing analysis. The results showed that, compared with that in the dominant bacterial species Ent_181 and Ent_189 of Group A, the expression of lignocellulose-decomposing enzymes was significantly downregulated in Bac_133 and Bac_71 (*p* < 0.05). In addition, we found that the dominant bacterial species of the tomato endophytic microbiome were more likely to become dominant populations in the wheat root microbiome. In general, our results demonstrated that lignocellulose-decomposing enzymes played a vital role in the formation of endophytes and their successful colonization of root tissues. This finding establishes a theoretical foundation for the development of broad-spectrum probiotics.

## Introduction

Plants harbor a set of taxonomically and functionally diverse microbial communities on their surfaces and within their tissues. Similar to the human microbiome, the plant microbiome contains an enormous number of genes, and the genetic content far exceeds that of the host plant itself (Yun, 2020). These microorganisms establish an intimate relationship with their hosts, ranging from beneficial or neutral interactions to harmful interactions (Bulgarelli et al., 2013; Turner et al., 2013; Nair and Padmavathy, 2014; Santoyo et al., 2016). In recent years, the plant microbiome has attracted much attention for its role in protecting the host against abiotic and biotic stresses, promoting plant nutrient uptake and utilization, protecting plants from pathogens, and improving plant health and production (Ma et al., 2011; Gaiero et al., 2013; Truyens et al., 2014; Hardoim et al., 2015; Blain et al., 2017; Iqbal et al., 2017). Plant microorganisms can directly antagonize pathogenic microorganisms through parasitism, secretion of antibiotics, or spatial nutrition competition and can indirectly inhibit microbial pathogenicity by inducing plant resistance or biological control agent (BCA) activity. The microbial communities associated with plants are regarded as one of the important functional driving forces for these eukaryotic hosts and can extend the host genome and metabolic capacity, providing or promoting a range of basic life-sustaining functions, including nutrient acquisition, immune regulation, and biological stress resistance (Cordovez et al., 2019). However, the application of beneficial bacteria in the field cannot achieve the expected results in practice. One reason for this is that not all beneficial bacteria have the ability to successfully colonize the plant rhizosphere or root tissue and play a role in biocontrol (Zhou et al., 2018). This phenomenon may be due to insufficient settlement in the rhizosphere or plant tissue, as the introduced microorganisms are usually washed away and do not survive at a meaningful functional density in plants or soil ecosystems. In this context, it is essential to understand the common pathways associated with the mechanisms of the assembly, activity, and persistence of the plant-associated microbiota and the interactions among them (Weller, 1988; Gibbons et al., 2016; Maldonado-Gomez et al., 2016; Sessitsch et al., 2018; Cordovez et al., 2019).

Studies showed that the assembly of the plant microbiome is a continuous and multistep process that is jointly determined by active diffusion, species interactions, the environment, and the host. Early colonizing microorganisms can be transmitted vertically through parental seed transmission routes. Once seeds germinate, the assembly of microorganisms is likely to be driven by horizontal transfer. Seed-borne microorganisms preferentially combine with aboveground plant tissues, while soil-derived microorganisms mainly combine with the rhizosphere and roots (Trivedi et al., 2020). The root microbiome is likely to be dynamically recruited and assembled during the life cycle of its plant host (Trivedi et al., 2020). A two-step selection model for root microbial flora differentiation has been previously reported. In this model, during the first step of differentiation, the characteristics of the root sediment and host cell wall promote the growth of bacteria that use organic nutrients, which lead to the migration of soil biological communities and the formation of rhizosphere communities. Taking strains of *Pseudomonas* as an example, when these strains come in contact with the plant root surface, they first form a colony and then form biofilms. In this way, *Pseudomonas* strains can colonize host plants as well as the fungi in the rhizosphere (Yang et al., 2010). During the second step of differentiation, the community structure of the rhizosphere and root is finely tuned. A comparison of the bacterial and fungal root microflora of mature poplar in two natural sites showed that the endophytic community composition was significantly different compared with that of the surrounding rhizosphere (Gottel et al., 2011), potentially because not all rhizospheric bacteria can become root endophytes (Bulgarelli et al., 2012; Tian et al., 2015). Due to host genetic factors and the selective colonization resistance of the host’s inherent microflora, only a small number of microorganisms are successful in colonization. Through verification of *Pseudomonas aeruginosa* (*P. chlororaphis*) strain PCL1391, Yang et al. (2010) found that mutant strains lacking colonization ability completely lost the ability to control damping off in tomatoes compared with the wild-type strains. The relationship between the colonization level of biocontrol strains of *P. fluorescens* in different parts of wheat roots and the number of spots of wheat take-all disease was studied, and the results confirmed that the colonization level of the biocontrol strains was inversely proportional to the number of disease spots on the host plants. The higher the level of colonization was, the lower the number of disease spots. When the root colonization level reached 10^7^–10^8^ CFU/cm, almost no disease spots were produced (Yang et al., 2010).

Colonization is a key step for limiting the biocontrol effect of beneficial microorganisms in plants (Yang et al., 2010). In general, the colonization ability of bacteria in host plant roots is related mainly to the genetic characteristics of the bacterial strains. First, microorganisms can attach themselves to plant surfaces for successful colonization, which is achieved through flagella and fimbriae (de Weert et al., 2002; Anna et al., 2017). The second step involves bacterial chemotaxis. de Weert et al. (2002) demonstrated the role of chemotaxis in the colonization ability of *Pseudomonas fluorescens* by constructing mutants of cheA, a key gene involved in the process of chemotaxis. Compared with that of the wild type, the colonization ability of the four mutants was weakened in all parts of the root, and this weakening increased from the root base to the root tip, indicating that chemotaxis played a very important role in the process of competitive site colonization. Third, a study showed that polymer-degrading enzymes, such as endoglucanase and polygalacturonidase, play an important role in helping bacteria penetrate into the endosphere of the root (Compant et al., 2005). Bulgarelli et al. (2012) and Xl et al. (2020) proposed that the lignocellulosic properties of plant hosts play a more important role than the internal environment of plants in determining whether rhizospheric bacteria become endophytes. There is evidence that colonization by endophytes in the internal tissues of plants involves the production of cellulases and pectinases, such as endoglucanase, pectinate lyase, and polygalacturonase (Alden et al., 2001). Thus, cell wall–degrading enzymes are most likely the key determinants of the initial entry and colonization by bacteria in plant hosts, and natural endophytic bacteria have the ability to secrete a variety of enzymes that help them penetrate the polysaccharide barrier, enabling them to survive in plants. After successful colonization by plant bacteria, the amount of bacteria colonizing the rhizosphere soil or plant roots plays a vital role in the biocontrol or beneficial effect of the rhizobacteria or endophytes.

In this study, 55 bacterial strains with plant growth-promoting effects were screened from isolated tomato endophytes and then used to inoculate wheat seeds. High-throughput sequencing and transcriptome sequencing were used to explore the following aspects: (1) examine the effect of bacterial inoculation with a single bacterium or a mixed bacterial complex on the community assembly and composition of the root endophytic microbiome of plants; (2) identify the factors that determine whether a microorganism becomes a root endophyte or successfully colonizes the inner tissue of plant roots; and (3) identify the reasons for the differences in colonization ability, which is why some strains exhibit colonization or even become excellent endophytes. A better understanding of the colonization ability of bacterial endophytes will contribute to the study of plant–endophyte interactions in agroecosystems and natural ecosystems.

## Materials and methods

### Isolation and identification of endophytic bacterial strains from tomato roots

The tomato cultivar Xinzhongshu No. 4, which was grown in a greenhouse under natural light conditions, was used for the isolation of endophytic bacteria. After 55 days of growth with nutrients, the tomato plants were pulled from the soil, and their roots were shaken to remove large soil particles. The harvested roots were carefully rinsed with tap water to remove the tightly attached soil, separately placed in 75% ethanol solution for 1 min and 5% NaClO solution for 3 min for sterilization, and then rinsed again with sterile water three times. The sterilized tomato roots were placed on LB agar plates at 37°C overnight to assess sterility to ensure that the isolated bacteria were from endophytic bacteria within the roots. The surface-sterilized root tissues were ground, serially diluted to 10^-7^, inoculated on different media, and incubated in an incubator at 28° or 37°C. The experimental medium included Luria-Bertani agar (LB), nutrient agar (NA), tryptic soy agar (TSA), Gauze No. 1 medium, and Hoagland nitrogen-free medium. The bacterial colonies on the plate were selected according to size, color, and shape. The isolated endophytic bacteria were tested for growth on LB at 28° or 37°C and transferred to a new plate two times for purification. Then, the purified strains were divided into two portions for seed preservation and stored at 20% glycerol in a -20°C freezer for further study.

The genomes of the isolated bacterial strains were extracted according to instructions for the Ezup Column Bacteria Genomic deoxyribonucleic acid (DNA) Purification Kit. A pair of universal primers, 27F and 1492R, was used for PCR amplification of bacterial 16S rDNA, using the extracted DNA as a template. The basic conditions for amplification were as follows: denaturation at 94°C for 30 s, followed by 30 cycles of annealing at 60°C for 30 s, and extension at 72°C for 90 s. The reaction volume was 50 μL, and 2 μL of DNA template was added for each reaction. The concentration of the PCR products was determined by 1% agarose electrophoresis. The purified PCR products of the 16S rRNA gene for each strain were sent for sequencing and molecular identification. After removing the repetitive strains, 55 isolated endophytic strains were selected for the subsequent bacterial colonization experiments, and the results of the molecular identification are displayed in Table 1.

**TABLE 1:** Taxonomic and lignocellulose identification of 55 endophytic strains isolated from tomato root microbiota.

Strains	Molecular identification	Sequence Identity (%)	Gene IDs	Xylanase activity	Cellulase activity
Ach_45	*Achromobacter insuavis*	100	ON242109	+ ++	+++
Aci_112	*Acidovorax monticola*	98.5	ON242156	+	+
Bac_1	*Bacillus velezensis*	100	ON242110	++	++
Bac_14	*Bacillus amyloliquefaciens*	99.93	ON242115	−	−
Bac_27	*Bacillus nitratireducens*	99.72	ON242122	+	+
Bac_36	*Bacillus subtilis*	99.79	ON242123	+	+
Bac_43	*Bacillus subtilis*	99.93	ON242124	−	+
Bac_44	*Bacillus cereus*	99.86	ON242125	−	+
Bac_51	*Bacillus cereus*	99.39	ON242126	+ ++	+++
Bac_64	*Bacillus aryabhattai*	100	ON242127	+ +	++
Bac_67	*Bacillus megaterium*	99.86	ON242128	+ +	++
Bac_68	*Bacillus megaterium*	99.57	ON242129	−	−
Bac_71	*Bacillus cereus*	99.79	ON242130	+ ++	+++
Bac_79	*Bacillus cereus*	100	ON242131	+ ++	+++
Bac_92	*Bacillus aryabhattai*	99.56	ON242132	+ +	++
Bac_98	*Bacillus subtilis*	99.78	ON242133	+ +	++
Bac_101	*Bacillus velezensis*	99.16	ON242111	+ +	++
Bac_133	*Bacillus subtilis*	99.86	ON242112	+ +	++
Bac_138	*Bacillus cereus*	99.79	ON242113	+ ++	+++
Bac_139	*Bacillus cereus*	96.68	ON242114	+ ++	+++
Bac_152	*Bacillus anthracis*	99.65	ON242116	+ ++	+++
Bac_165	*Bacillus velezensis*	99.29	ON242117	+ +	++
Bac_183	*Bacillus altitudinis*	100	ON242118	+ +	++
Bac_186	*Bacillus paranthracis*	99.7	ON242119	+ ++	+++
Bac_204	*Bacillus subtilis*	100	ON242120	+ +	++
Bur_95	*Burkholderia ambifaria*	99.84	ON242134	−	−
Ent_2	*Enterobacter ludwigii*	99.43	ON242141	+	+
Ent_4	*Enterobacter ludwigii*	99.58	ON242147	++	++
Ent_15	*Enterobacter mori*	99.26	ON242136	−	+
Ent_17	*Enterobacter cloacae*	99.03	ON242137	+	+
Ent_22	*Kosakonia oryzendophytica*	99.93	ON242142	−	−
Ent_29	*Enterobacter cloacae*	98.78	ON242143	−	−
Ent_33	*Enterobacter ludwigii*	99.56	ON242144	+	+
Chr_38	*Chryseobacterium sediminis*	99.55	ON242145	−	+
Ent_39	*Enterobacter* sp.	99.79	ON242146	−	+
Ent_113	*Enterobacter cloacae*	100	ON242135	+	+
Ent_117	*Enterobacter quasiroggenkampii*	96.74	ON242152	++	++
Ent_181	*Enterobacter cloacae*	99.71	ON242138	+	+
Ent_188	*Enterobacter cloacae*	100	ON242139	+++	+++
Ent_189	*Enterobacter cloacae*	100	ON242140	+	+
Lel_129	*Lelliottia amnigena*	99.46	ON242148	−	−
Lys_159	*Lysinibacillus xylanilyticus*	99.44	ON242149	+	−
Pan_120	*Pantoea*_sp.	99.86	ON242150	−	−
Pse_104	*Pseudomonas frederiksbergensis*	99.48	ON242151	+ ++	+++
Pse_61	*Pseudomonas plecoglossicida*	99.72	ON242153	+ +	++
Pse_77	*Pseudomonas plecoglossicida*	99.78	ON242154	+ ++	+++
Pse_97	*Pseudomonas extremaustralis*	98.67	ON242155	+	+
Pse_208	*Pseudomonas nicosulfuronedens*	99.43	ON242121	++	++
Rhi_114	*Rhizobium radiobacter*	99.02	ON242157	+	+
Rhi_130	*Rhizobium larrymoorei*	99.84	ON242158	+	+
Rhi_34	*Rhizobium radiobacter*	99.34	ON242159	+	+
Ser_99	*Serratia GRIMESII dsm*	99.78	ON242160	−	−
Sta_52	*Staphylococcus pasteuri*	99.93	ON242161	+ +	++
Sta_72	*Staphylococcus epidermidis*	99.93	ON242162	+ +	++
Ste_26	*Stenotrophomonas maltophilia*	99.41	ON242163	+	+

+ means enzymatic activity, −means no enzymatic activity.

+ (Within 0.5 cm of hydrolysis circle), + + (within 0.5 1.9 cm), + + + (above 2 cm).

### Screening of the cellulase and xylanase activities of the isolated bacterial endophytes

In this experiment, the cellulase and xylanase activities of 55 isolated bacterial strains were detected. To screen for cellulase activity, the overnight cultured bacterial solutions were spotted on CMC agar plates (0.2% NaNO_3_, 0.1% K_2_HPO_4_, 0.05% MgSO_4_, 0.05% KCl, 0.2% carboxymethyl cellulose (CMC), 0.02% peptone, and 1.7% agar in 1,000 mL of H_2_O) with a sterile tip. The CMC agar plates were incubated at 28°C for 48 h and stained with Gram’s iodine solution (2.0 g of KI and 1.0 g of iodine in 300 ml of distilled water) for 3–5 min. The cellulase activity was determined by observing the presence or absence of hydrolytic circles. Standard cellulose solution was used as a positive control, and sterile water was used as a negative control. To screen for xylanase activity, similar agar plates were prepared using xylan as a substrate rather than CMC, and then, the plates were incubated at 28°C for 48 h and stained with Gram’s iodine solution for 3–5 min.

### Bacterial inoculation and colonization experiment with wheat plants

For the single-bacterial inoculation experiment, the 55 endophytic strains were separately inoculated in liquid LB medium and cultured at 28°C (120 r ⋅ min^-1^) for 8 h (some strains were shaken for 10–24 h). The concentrations of the bacterial cultures were determined at an OD_600_ of 0.5, and 100 μL of bacterial suspension was collected for each strain. Wheat seeds were surface-sterilized with NaClO (5%) for 10 min and thoroughly rinsed with sterile water three times. The sterilized seeds were sown in sterile plastic chambers with 2–3 layers of filter paper and incubated overnight at 28°C under dark conditions. Ten healthy wheat seeds were then placed in sterile chambers with 2–3 layers of filter paper and were separately treated with 50 μL of bacterial culture suspension. All the treated samples were transferred into a light incubator at 28°C for 3–5 days and then incubated under natural light at room temperature for 10–12 days. Sterile water was added promptly to keep the filter paper moist. The control plants were treated with a sterile medium solution. The experiment for each bacterial strain was carried out in triplicate.

After the single-bacterial inoculation experiment for the 55 strains, we selected 4 bacterial strains with the different ability to colonize wheat roots, Ent_181 (absolute dominance), Ent_189 (absolute dominance), Bac_71 (relative dominance), and Bac_133 (non-dominance), according to their abundance in the inoculated root samples. Furthermore, we constructed 3 mixed bacterial complexes using the 4 selected bacterial strains and their taxonomic relatives (Table 2). Based on these 4 strains and the 3 mixed bacterial complexes, we compared the effects of single- and mixed-bacterial inoculation on the root endophytic microbiome in wheat. The experiment with 4 single-bacterial and 3 mixed-bacterial inoculations was performed using the same protocol as that described above.

**TABLE 2:** Bacterial strains of the inoculated bacterial complex in the wheat root.

Groups	Bacterial strain	Taxonomy	Abundance status
Single	Bac_71	*Bacillus*	Dominant bacteria
	Bac_133	*Bacillus*	Non-dominant bacteria
	Ent_181	*Enterobacter*	Dominant bacteria
	Ent_189	*Enterobacter*	Dominant bacteria
BacM	Bac_71	*Bacillus*	Dominant bacteria
	Bac_133	*Bacillus*	Non-dominant bacteria
	Bac_64	*Bacillus*	Dominant bacteria
	Bac_79	*Bacillus*	Dominant bacteria
	Bac_68	*Bacillus*	Dominant bacteria
	Bac_27	*Bacillus*	Non-dominant bacteria
	Bac_138	*Bacillus*	Non-dominant bacteria
	Bac_139	*Bacillus*	Non-dominant bacteria
	Bac_186	*Bacillus*	Non-dominant bacteria
EntM	Ent_181	*Enterobacter*	Dominant bacteria
	Ent_189	*Enterobacter*	Dominant bacteria
	Ent_113	*Enterobacter*	Dominant bacteria
	Ent_29	*Enterobacter*	Dominant bacteria
	Ent_17	*Enterobacter*	Dominant bacteria
Mix	Bac_71	*Bacillus*	Dominant bacteria
	Bac_133	*Bacillus*	Non-dominant bacteria
	Bac_64	*Bacillus*	Dominant bacteria
	Bac_79	*Bacillus*	Dominant bacteria
	Bac_68	*Bacillus*	Dominant bacteria
	Ent_181	*Enterobacter*	Dominant bacteria
	Ent_189	*Enterobacter*	Dominant bacteria
	Ent_113	*Enterobacter*	Dominant bacteria
	Ser_99	*Serratia grimesii*	Dominant bacteria
	Bur_95	*Burkholderia*	Dominant bacteria

### Genomic deoxyribonucleic acid extraction for the wheat root samples with single- and mixed-bacterial inoculations and Illumina high-throughput sequencing

The healthy wheat plants were separately collected for each treatment, and roots were removed with sterile scissors. Wheat roots were disinfected in 5% NaClO solution for 30 s with sterilized forceps and then rinsed with sterile water three times. Subsequently, they were disinfected with 75% ethanol for 5–10 s and rinsed with sterile water three times again. The sterilized roots were used for the extraction of total genomic DNA by using the PowerSoil^®^ DNA Isolation Kit (MoBio Laboratories, Carlsbad, CA, United States) according to the instructions. The extracted total genomic DNA was stored at –80°C until being subjected to high-throughput sequencing.

DNA fragments of the V5–V7 region of the bacterial 16S rRNA gene were amplified using the primer pair 799F (AACMGGATTAGATACCCKG) and 1193R (ACGTCATCCCCACCTTCC) and fused with Illumina MiSeq adapters and a 6-bp barcode sequence unique to each sample. PCR amplifications were carried out in triplicate in a 50-μL reaction system using the extracted genomic DNA as templates. The PCR amplification products were subsequently purified, combined in equimolar ratios, and subjected to high-throughput sequencing with the Illumina MiSeq sequencing platform, which produced 250 paired-end nucleotide reads, at Sangon Biotech (Shanghai, China). Two rounds of sequencing were conducted to ensure that an adequate amount of bacterial DNA (no less than 4,000 reads for each sample) was available for subsequent analysis after the removal of all the chloroplast sequences.

### Data processing and statistical analysis of the endophytic microbiome of wheat roots inoculated with bacteria

The raw sequences were separated according to barcode tags, and pairs of short-read sequences (reads) were spliced with FLASH software (version 1.2.3). Barcodes and primer sequences were removed using Cutadapt (version 1.9.1). Low-quality sequences with ambiguous bases, average quality scores < 25, or sequences shorter than 200 bp were removed to control sequence quality. Then, chimeric sequences were identified and removed with a *de novo* method using USEARCH (version 8.1.1861) (Edgar, 2010). After sequence control, the obtained effective sequences were clustered into operational taxonomic units (OTUs) at 97% sequence identity using Usearch (version 8.1.1861). The sequence with the highest abundance in each OTU was selected as the representative sequence. The obtained representative sequences were compared and annotated with the SILVA database using the QIIME package RDP Classifier to obtain the taxonomic information corresponding to each OTU (Haas et al., 2011). According to the obtained classification information of the OTUs, the OTUs classified as plant chloroplast and mitochondrial were deleted from the representative sequence. New OTU clustering and classification annotation were carried out to generate OTU tables for subsequent data analysis. To ensure the consistency of the results, a fixed number of sequence reads from each sample were separately rarefied on the bassi of the sample with the smallest number of reads. OTU clustering and classification analysis were then performed to generate OTU tables for subsequent data analysis.

The taxonomic units and their relative abundances in each sample were visualized by drawing bar charts and heatmaps based on the number of reads using the R package gplots (version 2.17.0). The diversity indexes (Shannon and Simpson index) and species richness estimators (Chao1 diversity and ACE index) for each sample with respect to a sequence depth of 3% were calculated using QIIME (version 1.8.0). Rarefaction and rank abundance curves were generated at a 97% OTU similarity level. Principal co-ordinates analysis (PCoA) and hierarchical cluster analysis in QIIME were used to evaluate the beta diversity of samples and the similarities and differences in community composition of different samples. Statistical analysis was performed based on the unweighted UniFrac distances and Bray–Curtis matrix to determine the significant differences between samples. The differences in bacterial taxa among the groups of samples were identified by LEfSe (LDA effect size) analysis (Edgar, 2013). First, the species with significant differences in relative abundance between different treatments were calculated using the R package edgeR with a *p*-value less than 0.05 (and FDR to control the false-positive rate at less than 5%). Through statistical analysis of the degree of influence of species on the samples, the value of the influence of each significantly different species on the sample was obtained, and significantly different microorganisms with a value of influence greater than two could be visualized.

### Metatranscriptomic analysis for wheat root samples inoculated with bacterial strains

To reveal the potential functional traits corresponding to the bacterial localization in the wheat root microbiome, four endophytic strains, Ent_181, Ent_189, Bac_71, and Bac_133, with colonization ability were selected for inoculation into wheat roots in sterile Petri dishes. The inoculated plants were grown, and the roots were harvested as described above. After surface disinfection, the sterilized plant roots were cutoff and placed into sterile bowls for grinding. Then, Phosphate Buffer Saline (PBS) was added to dilute the grinding solution to prepare the bacterial suspension. The bacterial suspension was obtained by serially filtering through 100, 20, and 11 μm membranes to remove plant tissues. Finally, the obtained endophytic bacterial precipitates for each sample were collected and sent to a commercial service for RNA extraction and transcriptional sequencing by using Illumina HiSeq™.

The obtained raw data for each sample were first evaluated by FastQC, and then, reads containing adapters and low-quality bases (Q-score ≤ 5) were removed to obtain clean data. The clean data were assembled into transcripts *de novo* (Tjaden, 2015) and then mapped to the gene-coding sequences by using Bowtie2. The read counts were normalized to the gene lengths, and the total number of non-rRNA metatranscriptomic reads was converted to reads per kilobase of transcript per million reads mapped (RPKM) values for subsequent statistical analysis (Langmead and Salzberg, 2012; Wang et al., 2012). The collected assembled transcripts were annotated using blastx against the NCBI nr database. The top hits assigned to amino acid sequences with taxonomic information were then used for downstream analysis.

According to the annotation results for the transcripts, the annotated GeneOntology (GO) function information was obtained, and the top GO functions were used for the analysis of GO enrichment to draw the network diagram. KAAS was used to obtain the annotated Kyoto Encyclopedia of Genes and Genomes (KEGG) pathway information of the transcripts, and clusterProfiler was used for the analysis of the KEGG pathways and COG enrichment (Kanehisa and Goto, 2000; Tatusov et al., 2000; Yuki et al., 2007). Salmon was used to calculate the expression levels of gene, and WGCNA was used for the analysis of gene coexpression levels. Analysis of the expression of differential gene was performed using DESeq2 (Tatusov et al., 2000).

## Results

### Sequencing data statistics for the root endophytic microbiome of wheat infected with a single-bacterial strain or mixed bacteria

The total genomic DNA of wheat root samples infected with 55 single-bacterial strains, parallel control samples, and mixed bacteria was amplified and sequenced in the V5–V7 region of bacterial 16S rDNA using Illumina MiSeq with 2 × 250 bp reads. The obtained raw sequence reads were separately merged and filtered for quality control (removal of primer and vector sequences, sequences with low-quality scores, chimeric sequences, etc.). Finally, a total of 23,36,079 high-quality sequences for 58 root samples were obtained. On average, 41,713 high-quality sequences were obtained per sample (min = 27,871, max = 67,899). For the inoculation experiment with the mixed bacterial complex, 806,643 pairs of raw sequences (reads) were obtained for 21 root samples from 7 groups after high-throughput sequencing. These sequences were also filtered for quality control, and 690,046 high-quality sequences were obtained. On average, 32,859 high-quality sequences were obtained per sample (min = 32,206, max = 33,291).

### Identification of wheat root endophytes and the effects of single-bacterial inoculation on the community structure and composition of the wheat root microbiome

**FIGURE 1:**
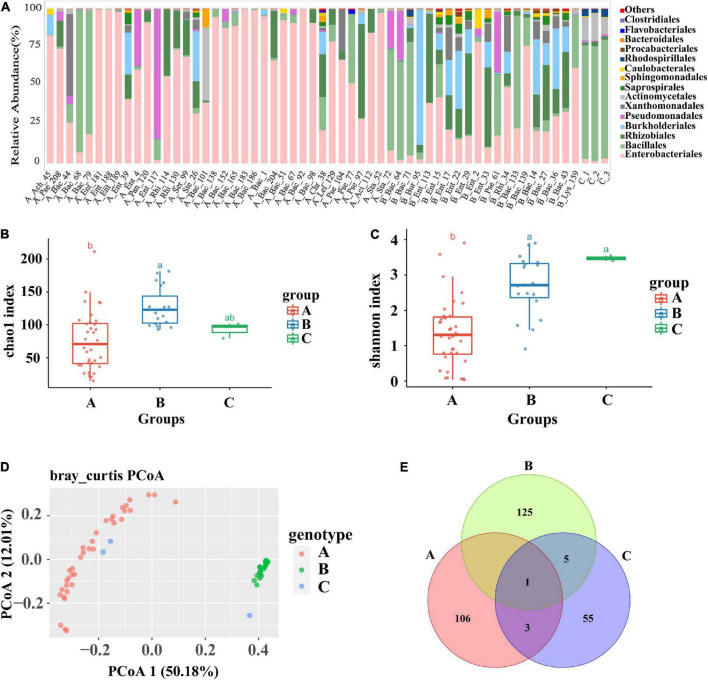
Analysis of the composition and diversity of the flora of the roots of tomato infested with 55 single bacteria and the control group. **(A)** Diagram of the species structure of 55 single bacteria and the control group (at the order level, selecting the top 15 flora in terms of abundance). **(B,C)** The Chao1 boxplot and Shannon boxplot of 55 single bacteria and control group (the letters above represent the differences between groups). **(D)** PCoA plots of 55 single bacteria and the control group, principal coordinated analysis (PCoA) derived from dissimilarity matrix of Unweighted UniFrac distance. **(E)** A Venn plot of 55 single bacteria and the control group. Among them, 55 single bacteria were divided into group A and group B due to the difference in the diversity of root flora.

The 55 identified plant growth-promoting bacterial strains belonged to 13 bacterial taxa, including *Achromobacter*, Bacillaceae, *Chryseobacterium*, *Lysinibacillus*, *Pantoea*, Pseudomonadaceae, *Acidovorax*, Rhizobiaceae, *Serratia*, *Staphylococcus*, *Stenotrophomonas*, and *Burkholderia*, covering a broad range of bacterial phyla. The wheat root samples with single-bacterial inoculation were divided into Group A, Group B, and the control group for further bioinformatic analysis according to the Shannon index and community diversity of the endophytic microbiome. Compared with those of the control wheat group, the community structure and the composition of the root endophytic root microbiome of the inoculated groups changed greatly, suggesting that single-bacterial inoculation greatly affected the structure and proportion of the flora in the wheat roots (Figure 1A). In the control samples, Bacillales (74.9%) was the predominant bacterial group of the wheat root microbiome, followed by Actinomycetales (14.3%), Rhodospirillales (4.12%), and Enterobacteriales (2.49%). In contrast, Bacillales was not the dominant bacterial group in most wheat root samples with single-bacterial inoculation (Figure 1A). Moreover, the OTU richness and bacterial diversity of the root microbiome were significantly different between the control samples and the root samples with bacterial inoculation (*p* < 0.05), suggesting that single-bacterial inoculation had a significant effect on the bacterial diversity (Figures 1B,C). The grouping results showed that the diversity and abundance of the bacterial flora of Group A, Group B, and the control group were significantly different due to inoculation. Regardless of whether the corresponding bacterial strains were inoculated, Group A, which included 36 root samples, was mostly colonized by a certain type of flora, mainly the pathogenic bacterium of the genus *Enterobacter*. Compared with those of Group A, the root samples of Group B were colonized by more abundant and diverse bacterial communities. The Chao1 and Shannon plots showed significant differences in OTU richness and bacterial diversity between Group A and Group B. The OTU richness and bacterial diversity of the flora within Group B were significantly higher than those of the flora within Group A, which was consistent with the results of the histogram analysis for single bacteria (*p* < 0.01) (Figures 1B,C). Simultaneously, the PCoA diagram showed that the root samples were significantly separated into two different groups, i.e., Group A and Group B, and both of them were significantly different from the control (Figure 1D; ANOSIM: *R* = 0.302, *p* = 0.001). The result was further verified by a Venn diagram, in which only a few OTUs were shared among the three groups or between pairs of samples (Figure 1E). In summary, single-bacterial inoculation significantly affected the community assembly and composition of the endophytic microbiome in wheat. In most cases, single-bacterial inoculation resulted in a lower abundance and diversity of endophytic communities in plant roots and a higher proportion of pathogenic bacteria.

### Identification of endophytic strains that colonized wheat roots

**FIGURE 2:**
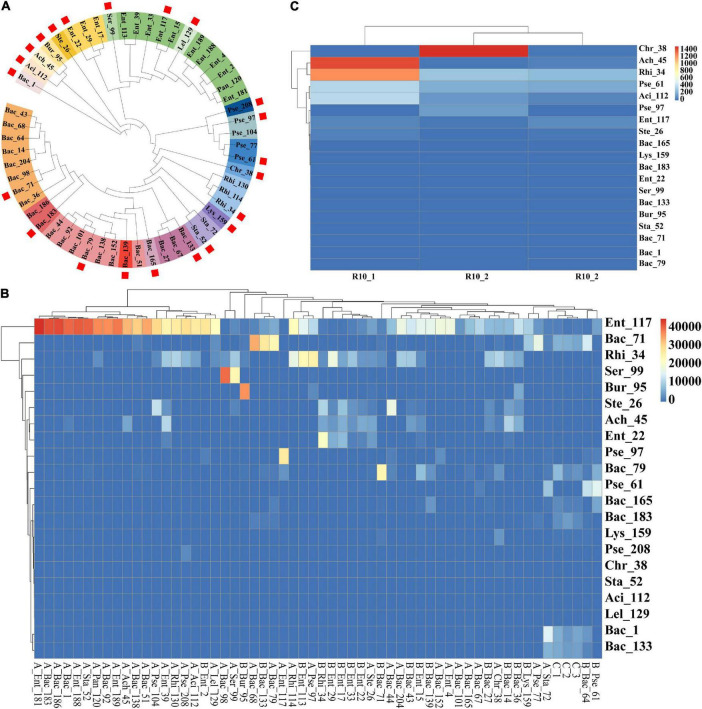
High-throughput sequencing of wheat and healthy tomato and heatmap of 55 single bacteria. High-throughput sequencing data of 55 infected wheat roots and the data of healthy tomato roots were mapped with a similarity of 97% with the representative sequences of 55 monocultures, respectively. **(A)** Screening dendrogram of representative sequences of 55 single bacteria. The representative sequences of the 55 single strains were screened, and each sample was blasted with the other samples. The similarity between the representative samples and the samples within the group was more than 99%, and the similarity between the samples within the group was more than 98%. A kind of color represents a set of representative sequences, and samples marked with squares are representative strains. **(B)** Heatmap of high-throughput data and representative sequences with 55 infected wheat roots. **(C)** High-throughput data within healthy tomato roots and heatmap of representative sequences.

Before identifying the endophytic strains that colonized wheat roots, bacterial strains with more than 99% sequence identity were first merged and regarded as one type of representative strain by pairwise alignment and the construction of a phylogenetic tree using the full-length 16S rDNA sequences of the 55 bacterial strains. Finally, 22 representative bacterial species with unique sequences were obtained (Figure 2A). To identify the endophytic strains with the ability to colonize wheat roots, high-throughput sequencing reads for 55 wheat root samples were separately mapped on the 22 representative sequences at a sequence identity threshold of 97%. The mapped reads were collected and visualized in a heatmap (Figure 2B). In the mapping results, the Bac_139 sample did not map to the corresponding strain, so there were 21 strains in the heatmap. The strains with more than 1,000 mapped reads were designated dominant bacterial species of the wheat root microbiome. Finally, 28 of the 55 bacterial strains were found to constitute the dominant bacterial group in the wheat root microbiome (Figure 2B). However, in most cases, bacterial inoculation did not always result in successful colonization or in the strain becoming the dominant population in the inoculated wheat roots. It is likely that the bacterial species belonged to the dominant bacterial taxa in the wheat root microbiome. To further compare the differences in the dominant bacterial populations between the tomato and wheat root microbiomes, we also downloaded the raw sequencing reads of the tomato root microbiome (wherein we isolated the 55 bacterial strains from the tomato plants) and mapped them onto the 22 representative sequences of type bacterial strains (Tian et al., 2015). Finally, a total of 8 bacterial strains were identified as the dominant population in the tomato root microbiome (Figure 2C). Among these, only 5 bacterial strains of the 55 strains, including Rhi_34, Rhi_114, Rhi_130, Ach_45, and Pse_61, were found to be dominant bacteria in both wheat and tomato roots. The results showed that a small portion of the dominant bacterial species coexisted in different plant species or that the dominant bacterial species were not always dominant in another plant. However, at a higher taxonomic level, most of the dominant bacterial orders in the tomato root microbiome, including Actinomycetales, Pseudomonadales, Rhizobiales, Burkholderiales, and Enterobacteriales, were also the dominant bacterial communities in the wheat root microbiome. This was also true at the bacterial phylum level (Tian et al., 2015).

### Identifying the main factors that affect bacterial colonization and community assembly in wheat roots

To identify the main factors that affect bacterial colonization and the community differences in the wheat root microbiome between the groups, a constrained analysis of principal components (CAP) with the environmental variables was performed (Supplementary Table 1). The results showed that lignocellulose-decomposing enzyme activities and the taxonomy of the inoculated bacterial strains were the main factors that affected the community assembly of and differences between Group A and Group B (ANOVA: *p* = 0.005). Among these, cellulase activity was significantly related to the root samples that harbored the dominant bacterial population in the endophytic microbiome (ANOSIM: *R* = 0.1358, *p* = 0.025), indicating that lignocellulose-decomposing enzymes play an important role in the mechanism of bacterial infection in plants. Among the 55 endophytic bacterial strains used in this study, most strains showed clear cellulase and xylanase activities (Table 1), indicating that they originated from the inner tissue of plant roots. A comparison of the enzyme activities of the bacterial strains from Group A and Group B showed that most of the strains from Group A had higher cellulase and xylanase activities than those of Group B, which might explain why wheat root samples from Group A always harbored the predominant bacterial population in the endophytic microbiome. In summary, lignocellulose-decomposing enzyme activity has a significant effect on the wheat root microbiome, which further verified the role of enzymes during the process of flora establishment. The lignocellulose decomposition ability was a key factor affecting inoculation with beneficial bacteria, for both bacterial localization in root tissue and assembly of the root endophytic microbiome.

### Effect of inoculation with a mixed bacterial complex on the community composition and bacterial diversity of the endophytic microbiome in wheat roots

**FIGURE 3:**
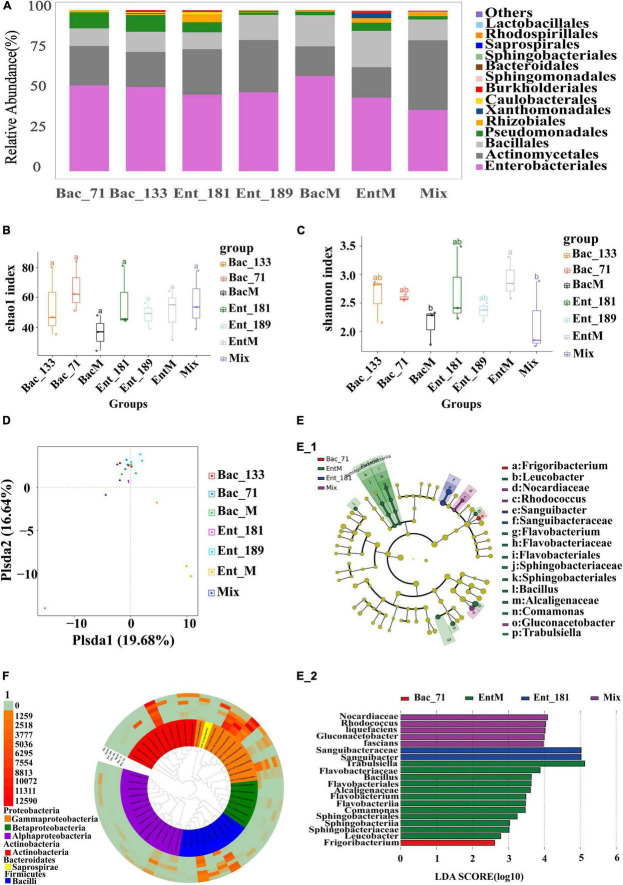
Secondary analysis of the composition and diversity of the flora of the roots of tomato after the infestation of four single and mixed bacteria. **(A)** Diagram of the species structure of 4 single bacteria and the mixed bacteria (at the order level, selecting the top 15 flora in terms of abundance). **(B,C)** The Chao1 boxplot and Shannon boxplot of 4 single bacteria and the mixed bacteria (the letters above represent the differences between groups). **(D)** PCoA analysis of single bacteria and the mixed bacteria, principal coordinated analysis (PCoA) derived from dissimilarity matrix of Unweighted UniFrac distance. **(E)** LEfSe difference analysis diagram of single bacteria and mixed bacteria (different circles represent different hierarchical classification, from inside to outside, followed by phylum, class, order, family, and genus. Each node represents a species, and yellow means that the species is not significantly different within several groups. Other colors indicate that there are differences in the corresponding samples, and the species with specific differences are marked on the right side). **(F)** Tree diagram with OTU high value of single bacteria and mixed bacteria (the top 50 OTUs in terms of abundance were selected to draw the tree diagram, and different colors represent different classes. The outer layer of the tree diagram is the abundance of OTUs, and different colors represent different abundances).

To further verify the effect of bacterial inoculation and inoculated bacteria on the community assembly and composition, we selected 4 bacterial strains from the 55 endophytic strains, Ent_181 (absolute dominance), Ent_189 (absolute dominance), Bac_71 (relative dominance), and Bac_133 (non-dominance), according to their abundance in the inoculated root samples. Furthermore, we constructed 3 mixed bacterial complexes using the selected 4 bacterial strains and their taxonomic relatives (Table 2). Based on these 4 strains and the 3 mixed bacterial complexes, we compared the effects of single- and mixed-bacterial inoculation on the root endophytic microbiome in wheat. Consistent with the previous inoculation experiment, inoculation with a single strain or single-bacterial species in wheat roots, including with Ent_181, Ent_189, Bac_71, Bac_133, mixed *Bacillus* strains (BacM), and mixed *Enterobacter* strains (EntM), resulted in an Enterobacteriales-dominant microbiome, with a proportion of 45–50% (Figure 3A and Table 2). In contrast, inoculation with the bacterial complex Mix, which included 10 different species from *Enterobacter*, Bacillus, *Serratia*, and *Burkholderia*, resulted in a lower abundance of Enterobacteriales, with Actinomycetales accounting for the highest proportion (Figure 3A and Table 2). Compared with single-bacterial inoculation, mixed-bacterial inoculation led to a more balanced diversity of flora composition, and the probability of the emergence of a single bacterium with absolute dominance was lower, especially for pathogenic bacteria (Figures 3B,C). However, among the four single-bacterial strains, the Bac strains were more diverse than the Ent strains, precisely as a result of the high abundance of *Enterobacter* colonization. There was also variability among the mixed bacteria, with differences in bacterial diversity observed between BacM and EntM and between EntM and Mix, probably due to the influence of the *Enterobacter* species. The *Enterobacter* strain corresponding to OTU3 was highly abundant in the Ent_189 and Ent_M samples. The PCoA results demonstrated that the root samples Bac_71, Bac_133, BacM, and Mix shared a higher community similarity, while the *Enterobacter*-inoculated samples had significant differences among themselves and were also different from the *Bacillus* and Mix samples (Figure 3D).

The results demonstrated that the inoculated *Enterobacter* strains could easily infect and colonize root systems *via* complex interactions. The *Enterobacter* strain corresponding to OTU101 had a relatively high abundance in Ent_181. Although the inoculated *Bacillus* strains were also found to be localized in wheat roots, such as the *Bacillus* strain corresponding to OTU5 in Bac_71 and that, corresponding to OTU4 in BacM, the abundance of the corresponding *Bacillus* OTUs was relatively low, and the infection was less effective than that with the *Enterobacter* strains (Figure 3A). We further identified the differences in the communities between the root samples by using LEfSe (Figures 3E,F). Most of the identified differentially abundant bacterial taxa were from Actinomycetales and Enterobacteriales, among which the difference in Enterobacteriales was the largest. The results seemed to be consistent with the analysis of community structure and composition, and *Enterobacter* strains were among the dominant colonizers.

### Identification of functional traits and genes corresponding to bacterial infection and localization in wheat roots using transcriptome analysis

**FIGURE 4:**
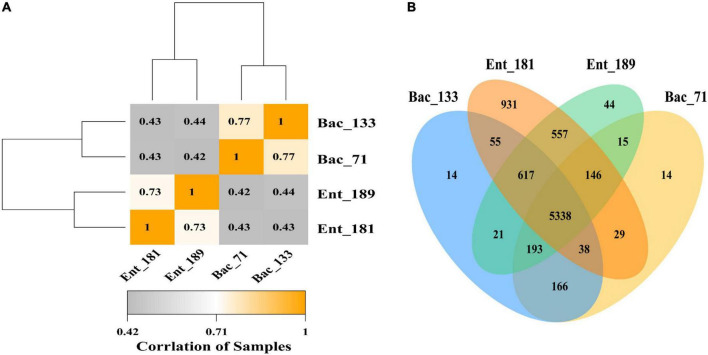
Transcriptome analysis of 4 significant colonizing bacteria. **(A)** Pearson correlation coefficient heat map of transcriptome expression of different samples. **(B)** A Venn map of gene expression between different samples.

To reveal the potential functional traits corresponding to bacterial localization and the main factor that affected community assembly in the wheat root microbiome, we performed transcriptomic sequencing for the samples inoculated with bacterial strains Ent_181, Ent_189, Bac_71, and Bac_133. The Venn diagram results showed that the number of specifically expressed genes in the *Enterobacter* samples Ent_181 and Ent_189 was higher than that in *Bacillus* samples Bac_71 and Bac_133, and the number of specifically expressed genes in the *Enterobacter* sample Ent_181 was 931 (Figure 4A). In addition, the results for the difference in the expression of gene between samples were also consistent with the results of the correlation analysis. The heatmap of the correlation analysis between samples showed that the correlation between Ent_181 and Ent_189 was relatively high, with a value of 0.73, while the correlations between Ent_181 and Bac_71 and between Ent_181 and Bac_133 were both 0.43, with good repeatability for each group of samples (Figure 4B). In the KEGG pathway analysis, we analyzed and compared the genes that were upregulated or downregulated during bacterial infection and their localization in the different samples (Table 3). The results showed that the levels of four pathways related to bacterial colonization, mainly starch and sucrose metabolism, bacterial chemotaxis, tryptophan metabolism, and flagellar assembly, were significantly different between samples. Studies showed that lignocellulose-decomposing enzymes might be involved in root colonization by bacterial flora, and there is evidence that colonization by endophytes in internal plant tissues involves the production of cellulase and pectinase (Reinhold-Hurek et al., 2006; Rosenblueth and Martínez-Romero, 2006). To gain a deeper understanding of the role of these enzymes, the expression levels of genes related to lignocellulose degradation were further compared. The results showed that α-amylase, beta-glucosidase, maltase-glucoamylase, beta-fructofuranosidase, alpha-trehalase, glucan 1,3-beta-glucosidase, 6-phospho-beta-glucosidase, beta-galactosidase, pectinesterase, and other genes encoding cell wall–degrading enzymes, acting mainly on cellulose and pectin, were identified (Table 4). Compared with the *Bacillus* sample, most of the identified lignocellulose genes were upregulated in *Enterobacter* samples Ent_181 and Ent_189 (Table 4). The transcriptomic analysis results verified that lignocellulose-decomposing enzymes might play an important role in bacterial colonization of plant roots, and the *Enterobacter* sample had higher expression levels of enzymes related to lignocellulose degradation, especially pectinases, which was consistent with the pathogenic trait of the *Enterobacter* strains (Rafique et al., 2021). In addition to the role of enzymes, the colonization by the Bac samples in plants might be related to bacterial chemotaxis and flagellar assembly, which illustrated the complexity of the colonization process.

**TABLE 3:** The identified differential expression level for the KEGG pathway using transcriptomic analysis.

Sample pair (*P*-value)	Starch and sucrose metabolism	Bacterial chemotaxis	Flagellar assembly	Tryptophan metabolism
Bac_71_vs_Ent_181.down	0.038***	0.59	0.99	0.07**
Bac_71_vs_Ent_181.up	0.89	0.07**	5.19E-08***	0.96
Bac_71_vs_Ent_189.down	0.000357315***	6.19E-05***	0.62172586	0.48
Bac_71_vs_Ent_189.up	0.56	0.98	0.19*	0.8
Bac_133_vs_Ent_181.down	0.08**	0.82	0.99	0.02***
Bac_133_vs_Ent_181.up	0.94	0.16*	7.54E-09***	0.87
Bac_133_vs_Ent_189.down	0.02***	0.000007 ***	0.102	0.23
Bac_133_vs_Ent_189.up	0.7	0.9	0.53	0.8
Ent_181_vs_Ent_189.down	0.97	0.006***	1E-11***	0.65
Ent_181_vs_Ent_189.up	0.93	0.99	no	0.07**
Bac_71_vs_Bac_133.down	0.4*	no	no	0.68
Bac_71_vs_Bac_133.up	0.72	0.92	0.8	0.26

Differential up- or downregulation of gene expression is reflected by the p-value.

*0.1 < p<0.5; **0.05 < p<0.1; ***p < 0.05.

**TABLE 4:** The identified differential expression level for the lignocellulose genes using the transcriptomic analysis.

EC	CAZymes	Bac_133_ vs_Ent_181	Bac_133_ vs_Ent_189	Bac_71_ vs_Ent_181	Bac_71_ vs_Ent_189	Bac_71_ vs_Bac_133	Ent_181_ vs_Ent_189
3.2.1.1	alpha-amylase	2_down	2_down	2_down	2_down	2_down	2_balance
3.2.1.20	maltase-glucoamylase	2_down	2_balance	2_down	2_down	2_balance	2_balance
3.2.1.21	beta-glucosidase	3_balance	3_balance	3_up	3_up	3_balance	3_balance
3.2.1.26	beta-fructofuranosidase	2__down	2_down	2_down	2_down	2_balance	2_up
3.2.1.28	alpha-trehalase	1_down	1_down	1_down	1_down	1_balance	1_up
3.2.1.58	glucan 1,3-beta-glucosidase	1_down	1_balance	1_down	1_balance	1_balance	1_up
3.2.1.86	6-phospho-beta-glucosidase	1_down	1_balance	1_down	1_balance	1_balance	1_balance

up, gene upregulation; down, gene downregulation; The numbers represent the number of occurrences of this enzyme gene.

### Analysis of the assembly process of the endophytic microbiome in wheat roots

**FIGURE 5:**
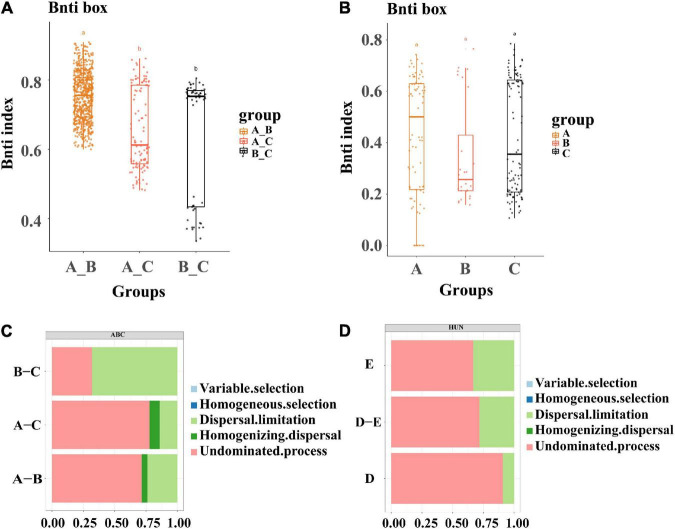
Analysis of microbiota assembly process. **(A)** Microbial assembly analysis of 58 single strains, divided into three groups of A, B, and C, which are viewed as control groups. **(B)** Microbial community assembly analysis of 4 single bacteria and mixed bacteria, where D represents the combination of four single bacteria and E represents the combination of three groups of mixed bacteria. **(C)** Analysis of the causes of community aggregation and composition of 55 single bacteria and the control group (divided into three groups of A, B, and C, which are viewed as control groups). **(D)** Analysis of the reasons for the aggregation and composition of single bacteria and mixed bacteria in the mixed bacteria test, where D represents the combination of four single bacteria and E represents the combination of three groups of mixed bacteria. The letters above the bars represent the differences between groups.

We studied the role of deterministic and stochastic assembly processes in the wheat root endophytic microbiome by calculating the relationship between βNTI and infection with different bacterial inoculation. The βNTI values of the 55 strains used for single-bacterial inoculation were divided into three groups: A, B, and C (Figure 5A). The results of pairwise comparisons showed that the differences in microbial populations among Group A, Group B, and the control group were mainly dominated by stochasticity (|βNTI| < 2). However, the differences between the two inoculation groups were significantly different from those between each of them and the control group; that is, bacterial inoculation had a significant effect on the community assembly and composition of the wheat root endophytic microbiome. Similar results were obtained for wheat roots inoculated with mixed bacterial complexes, which also showed that the community composition of the root endophytic microbiome was mainly dominated by stochasticity (Figure 5B). This result was consistent with those of studies showing that the community assembly process of soil- and root-associated microbiomes was initially controlled by random processes (Dini-Andreote et al., 2015).

In addition, we quantitatively estimated this stochastic assembly process. A comparison of bacterial inoculation between Group A and the control group showed that the undominant process was the most prevalent (Group A, 77%), and the same result was observed when comparing Group A with Group B (Group A, 71%), which indicated that the assembly process was mainly influenced by external factors; that is, inoculation had an effect on the assembly of the bacterial flora in wheat roots (Figure 5C). Among the mixed-bacterial inoculations, the non-dominant process of single bacteria of Group D (90%) was significantly higher than that of Group E (66%), which was related to the larger proportion of single bacteria in the single-bacterial inoculation group, and the strain composition of Group E was relatively balanced (Figure 5D). This result was also consistent with the results for the 55 strains of single bacteria, so it could be speculated that the establishment of the flora in the process of inoculation was mainly related to the non-dominant process.

## Discussion

Plant roots can release a large number of chemicals or organic compounds, such as amino acids, proteins, and organic acids, into soils to affect the microbial community of the rhizosphere and further affect the recruitment of bacterial endophytes in plant roots (Bulgarelli et al., 2012; Kawasaki et al., 2016; Pétriacq et al., 2017; Shyam et al., 2017). However, bacterial entry and localization in the root inner tissue are determined by many factors, such as bacterial chemotaxis, lipopolysaccharides, and flagella (Böhm et al., 2007). In addition, it is believed that endophytes can penetrate the root endodermis by secreting cell wall–degrading enzymes (CWDEs), including cellulase, xylanases, pectinases, and polygalactosidase, which enables them to continue to colonize and move in the endoderm (Lodewyckx et al., 2002; Compant et al., 2005; Sabbadin et al., 2021). In agriculture, the introduction of bacteria that are beneficial to plants is an important practice for improving plant productivity and stress resistance without the use of pesticides and inorganic fertilizers and for promoting the phytoremediation of heavy metals and hydrocarbons (Anna et al., 2017). Therefore, understanding the mechanism of bacterial colonization and the effect of bacterial inoculation on the community assembly and composition of the root endophytic microbiome will provide a basis for developing more successful applications for broad-spectrum probiotics.

With regard to microbial ecology, it has been hypothesized that microbial assembly is mainly dominated by deterministic and stochastic processes (Zhou et al., 2013). The stochastic process was mainly proposed based on neutral theory, which assumes that all species or individuals are ecologically absolutely equal, so community composition and distribution patterns are completely determined by stochastic processes. Many studies have demonstrated that stochastic processes drive the community assembly and composition of the rhizospheric microbiome in plants (Luan et al., 2020; Qiu et al., 2021). Few studies have been performed to reveal the community assembly process of the endophytic microbiome in plant roots. In this study, through βNTI analysis of the community differentiation of the root microbiome upon inoculation with single or mixed bacterial strains, we found that the community differences and composition of the root endophytic microbiome were also mainly dominated by stochastic processes (|βNTI| < 2). Compared with the control group, root samples with single-bacterial inoculation showed significantly different community assembly and composition, wherein the root samples of Groups A–B showed mainly non-dominant processes. Dispersal limitation (67%) was dominant in Groups B–C, which was related to the high diversity of Group B and the absence of single bacteria, further indicating that the flora differed during the process of inoculation. The results for the mixed bacteria and the single bacteria were basically consistent. The significant difference in the main factors in the assembly process of Group A and Group B of single bacteria was consistent with that in the diversity analysis of Group A and Group B. CAP analysis was performed on Group A and Group B, and the *p*-value in the combined model with cellulase activity was less than 0.05.

In the single-bacterial inoculation experiment for 55 strains, we found that infection by single bacteria was clearly dominated by 1–3 bacterial species with high abundance, and the dominant bacteria were most likely to be pathogenic bacteria, although these bacteria were also found to be the earliest colonizers among root endophytes in previous studies. In contrast, the community composition of root samples inoculated with the mixed bacterial strains was highly diverse and varied, with a relatively high abundance of Actinomycetales. These results were consistent with the ecological process analysis for the root endophytic microbiome; that is, bacterial inoculation significantly affected the community assembly of the root microbiome in plants. In addition, CAP analysis demonstrated that the lignocellulose-decomposing enzyme activities of the inoculated bacterial strains were among the main factors that affected the community assembly and composition. The further transcriptomic analysis also showed the significant differences in the expression of functional pathways and genes involved in lignocellulose degradation. It was then speculated that the formation of the flora did not simply follow the neutral theory but that there was a certain selective deterministic process (screened by the lignocellulose barrier) within the neutral theory. Hurek and Reinhold-Hurek (2003) found that mutation of the nitrogen-fixing bacterium BH72 by knocking out the genes encoding endo-β-1,4-glucanase (cellulase) led to the failure to invade the roots of rice, indicating that the production of some lignocelluloses may be necessary for bacterial strains to become endophytes. Further studies to validate the role of lignocellulose in bacterial localization in plant roots and to determine whether they could establish themselves in the plant environment after application as biological fertilizers or biocontrol agents in the field are needed.

## Data availability statement

The data presented in the study are deposited in the NCBI Short Read Archive, accession numbers SRX1489 1103–SRX14891181 and SRX14938537–SRX14938540 for community and transcriptomic analysis of the wheat root microbiome and the GenBank database, accession numbers ON242109–ON242163 for the full-length 16S rDNA of the isolated bacterial strains.

## Author contributions

BT and YZ designated and supervised the project. TZ, JX, and RT performed the experiments. TZ performed all the bioinformatic analyses and prepared figures and tables. BT, TZ, KL, and XX contributed to the manuscript’s writing and revision. YL, QZ, KL, XX, and LL suggested protocols, data analyses, and interpretation of results. All authors have read and approved the submitted version of the manuscript.
