# Supplementary material for: Effects of single- and mixed-bacterial inoculation on the colonization and assembly of endophytic communities in plant roots
Source: Front Plant Sci. 2022 Aug 29;13:928367. doi: 10.3389/fpls.2022.928367 (PMC9464981; doi:10.3389/fpls.2022.928367)
Supplement: Supplementary file 1 [file Data_Sheet_1.docx]

Supplementary Material

# Supplementary Tables

**Supplementary material Table S1 CAP analysis**

|  | Reads _level | groups | taxonomy | one_Xylanase_activity | one_Cellulase_activity | one_Enzyme_activity | two_Xylanase_activity | two_Cellulase_activity | two_Enzyme_activity | three_Xylanase_activity | three_Cellulase_activity | three_Enzyme_activity | chao1_1 | chao1_2 | shannon_1 | shannon_2 |
| --- | --- | --- | --- | --- | --- | --- | --- | --- | --- | --- | --- | --- | --- | --- | --- | --- |
| A_45 | E | A | Ach | high | high | high | high | high | high | high | high | high | low | low | low | no |
| A_101 | low | A | Bac | M | M | M | no | no | no | less | less | less | low | low | M | M |
| A_138 | low | A | Bac | high | high | high | high | high | high | high | high | high | high | high | M | low |
| A_152 | low | A | Bac | high | high | high | high | high | high | high | high | high | M | low | M | M |
| A_165 | low | A | Bac | M | M | M | no | no | no | less | less | less | high | high | M | low |
| A_183 | low | A | Bac | M | M | M | no | no | no | less | less | less | low | low | low | no |
| A_186 | low | A | Bac | high | high | high | high | high | high | high | high | high | low | low | low | no |
| A_1 | low | A | Bac | M | M | M | no | no | no | less | less | less | low | low | low | no |
| A_204 | low | A | Bac | M | M | M | no | no | no | less | less | less | high | high | M | low |
| A_208 | E | A | Pse | M | M | M | no | no | no | less | less | less | midle | low | M | low |
| A_44 | E | A | Bac | no | less | less | no | no | no | no | less | less | M | low | M | M |
| A_51 | low | A | Bac | high | high | high | high | high | high | high | high | high | high | high | M | low |
| A_67 | low | A | Bac | M | M | M | no | no | no | less | less | less | M | low | M | low |
| A_68 | E | A | Bac | no | no | no | no | no | no | no | no | no | high | high | low | no |
| A_79 | E | A | Bac | high | high | high | high | high | high | high | high | high | high | high | M | low |
| A_92 | low | A | Bac | M | M | M | no | no | no | less | less | less | low | low | low | no |
| A_98 | low | A | Bac | M | M | M | no | no | no | less | less | less | low | low | low | no |
| A_181 | E | A | Ent | less | less | less | no | no | no | less | less | less | low | low | low | no |
| A_188 | E | A | Ent | high | high | high | high | high | high | high | high | high | low | low | low | no |
| A_189 | E | A | Ent | less | less | less | no | no | no | less | less | less | M | low | low | no |
| A_38 | low | A | Chr | no | less | less | no | no | no | no | less | less | high | high | high | high |
| A_39 | E | A | Ent | no | less | less | no | no | no | no | less | less | high | high | M | M |
| A_4 | E | A | Ent | M | M | M | no | no | no | less | less | less | M | low | M | low |
| A_129 | low | A | Lel | no | no | no | no | no | no | no | no | no | M | low | M | low |
| A_77 | low | A | Pse | high | high | high | high | high | high | high | high | high | M | low | M | low |
| A_97 | low | A | Pse | less | less | less | no | no | no | less | less | less | high | high | M | low |
| A_112 | low | A | Aci | less | less | less | no | no | no | less | less | less | low | low | low | no |
| A_114 | E | A | Rhi | less | less | less | no | no | no | less | less | less | M | low | M | low |
| A_130 | E | A | Rhi | less | less | less | no | no | no | less | less | less | high | high | M | low |
| A_120 | E | A | Pan | no | no | no | no | no | no | no | no | no | low | low | low | no |
| A_104 | low | A | Pse | high | high | high | high | high | high | high | high | high | M | low | M | low |
| A_117 | E | A | Ent | M | M | M | no | no | no | less | less | less | low | low | low | no |
| A_99 | E | A | Ser | no | no | no | no | no | no | no | no | no | M | low | M | low |
| A_52 | low | A | Sta | M | M | M | no | no | no | less | less | less | low | low | low | no |
| A_72 | low | A | Sta | M | M | M | no | no | no | less | less | less | M | high | M | M |
| A_26 | E | A | Ste | less | less | less | no | no | no | less | less | less | high | high | high | high |
| B_133 | low | B | Bac | M | M | M | no | no | no | less | less | less | M | high | M | low |
| B_139 | low | B | Bac | high | high | high | high | high | high | high | high | high | high | high | M | M |
| B_14 | low | B | Bac | no | no | no | no | no | no | no | no | no | high | high | high | high |
| B_27 | low | B | Bac | less | less | less | no | no | no | less | less | less | high | high | high | high |
| B_36 | low | B | Bac | less | less | less | no | no | no | less | less | less | high | high | high | high |
| B_43 | low | B | Bac | no | less | less | no | no | no | no | less | less | high | high | high | high |
| B_64 | E | B | Bac | M | M | M | no | no | no | less | less | less | M | high | M | M |
| B_71 | E | B | Bac | high | high | high | high | high | high | high | high | high | high | high | M | M |
| B_95 | E | B | Bur | no | no | no | no | no | no | no | no | no | high | high | low | no |
| B_113 | E | B | Ent | less | less | less | no | no | no | less | less | less | M | high | M | low |
| B_15 | E | B | Ent | no | less | less | no | no | no | no | less | less | high | high | high | high |
| B_17 | E | B | Ent | less | less | less | no | no | no | less | less | less | high | high | high | high |
| B_22 | E | B | Ent | no | no | no | no | no | no | no | no | no | high | high | high | high |
| B_29 | E | B | Ent | no | no | no | no | no | no | no | no | no | high | high | M | M |
| B_2 | E | B | Ent | less | less | less | no | no | no | less | less | less | high | high | M | low |
| B_33 | E | B | Ent | less | less | less | no | no | no | less | less | less | high | high | high | high |
| B_159 | low | B | Lys | less | no | less | no | no | no | less | no | no | M | high | M | M |
| B_61 | E | B | Pse | M | M | M | no | no | no | less | less | less | high | high | M | M |
| B_34 | E | B | Rhi | less | less | less | no | no | no | less | less | less | high | high | M | M |

Reads _level：According to the number of reads after mapping with the representative sequence, more than 1000 are dominant bacteria(E)；group：According to the diversity of strain Shannon, PCOA analysis was performed and divided into AB groups；one_Xylanase_activity，one_Cellulase_activity：In enzyme activity, + stands for less. ++ stands for M, +++ stands for high, - stands for no ；one_Enzyme_activity：add up one_Xylanase_activity and one_Cellulase_activity；two_Xylanase_activity，two_Cellulase_activity：In enzyme activity, +++ stands for high, the remaining stands for no ；two_Enzyme_activity：add up two_Xylanase_activity and two_Cellulase_activity；

three_Xylanase_activity，three_Cellulase_activity：In enzyme activity, +++ stands for high, ++ and + stands for less, - stands for no ；three_Enzyme_activity：add up three_Xylanase_activity and three_Cellulase_activity；chao1_1: Chao1 index above 100 is high, 50-100 is M, and below 50 is low; chao1_2: Chao1 index above 93 is high, below 93 is low; shannon_1: Shannon index above 3 is high, 1-3 is M, 0-1 is low; shannon_2: Shannon index above 3 is high, 2-3 is M, 1-2 is low, 0-1 is no

**Supplementary Material Table S2: Table of Diversity and Richness of 58 Samples**

| sample | shannon | chao1 | sample | shannon | chao1 |
| --- | --- | --- | --- | --- | --- |
| Ach_45 | 0.906537552 | 26 | Ent_15 | 3.288469 | 178.6 |
| Aci_112 | 0.791458252 | 45.5 | Ent_17 | 3.219776 | 104.1667 |
| Bac_1 | 0.564784491 | 42 | Ent_181 | 0.059125 | 22.25 |
| Bac_101 | 2.500994855 | 36.5 | Ent_188 | 0.059376 | 26.33333 |
| Bac_133 | 1.718425107 | 97.8 | Ent_189 | 0.249284 | 93 |
| Bac_138 | 1.484670153 | 115 | Ent_2 | 1.573757 | 118.1429 |
| Bac_139 | 2.449862987 | 126.9 | Ent_22 | 3.845641 | 181.3684 |
| Bac_14 | 3.532055288 | 159.8824 | Ent_29 | 2.76321 | 123.2 |
| Bac_152 | 2.035523768 | 50.375 | Ent_33 | 3.388117 | 123.0667 |
| Bac_165 | 1.834983246 | 149.75 | Ent_39 | 2.95722 | 105.9091 |
| Bac_183 | 0.037071492 | 27 | Ent_4 | 1.264406 | 78.2 |
| Bac_186 | 0.092334537 | 15 | Lel_129 | 1.126556 | 62 |
| Bac_204 | 1.780315125 | 134.3333 | Lys_159 | 2.270462 | 96.06667 |
| Bac_27 | 3.903579669 | 164.0435 | Pan_120 | 0.798812 | 39 |
| Bac_36 | 3.279390948 | 109.5455 | Pse_104 | 1.262886 | 89.5 |
| Bac_43 | 3.361529381 | 167.75 | Pse_208 | 1.538134 | 71 |
| Bac_44 | 2.203184024 | 59 | Pse_61 | 2.713853 | 113.2308 |
| Bac_51 | 1.351726354 | 101.4286 | Pse_77 | 1.803441 | 65.16667 |
| Bac_64 | 2.689669675 | 99 | Pse_97 | 1.753833 | 104.1579 |
| Bac_67 | 1.663964738 | 76.33333 | Rhi_114 | 1.211675 | 70.6 |
| Bac_68 | 0.789347693 | 101 | Rhi_130 | 1.666164 | 133.4286 |
| Bac_71 | 2.480483033 | 127.5 | Rhi_34 | 2.468932 | 127.2174 |
| Bac_79 | 1.457265707 | 107.1111 | Ser_99 | 1.84451 | 88.3 |
| Bac_92 | 0.083425226 | 21 | Sta_52 | 0.286375 | 43.75 |
| Bac_98 | 0.68117637 | 47 | Sta_72 | 2.25975 | 94.6 |
| Bur_95 | 0.909170415 | 101.3636 | Ste_26 | 3.584415 | 135.625 |
| Chr_38 | 3.910094916 | 211.1714 | WS8_1 | 3.401877 | 97.2 |
| Ent_113 | 1.448145978 | 93.17647 | WS8_2 | 3.470694 | 79.66667 |
| Ent_117 | 0.900669161 | 38.66667 | WS8_3 | 3.545902 | 101.5714 |
